# Supplementary material for: Are carbon emissions trading and green financial instruments synergistic? -Comprehensive quantitative research based on content analysis
Source: PLoS One. 2024 Mar 7;19(3):e0298601. doi: 10.1371/journal.pone.0298601 (PMC10919601; doi:10.1371/journal.pone.0298601)
Supplement: S1 Data — (ZIP) [file pone.0298601.s001.zip › Data/地级市Z_F工作报告.zip!/╔╜╬≈/╨├╓▌╩╨/└┤╫╘╣1⁄2╓┌║┼í╛┬φ┐╦╩2╛▌═°í┐_2017.docx]

各位代表：

　　现在，我代表市人民政府向大会报告工作，请予审议，并请市政协委员和其他列席会议人员提出意见。

　　一、2016年工作回顾

　　过去的一年，面对艰巨繁重的工作任务和经济持续下行的困难局面，我们深入学习贯彻习近平总书记系列重要讲话精神，认真贯彻省委“一个指引、两手硬”重大思路和要求，按照省政府转型综改系列决策部署，全面实施市委“1661”发展战略，坚持“产业第一、项目至上、企业为重、服务为本”，立足自身努力，保持进取追求，统筹推进稳增长、促改革、调结构、惠民生、防风险等各项工作，在克服重重困难的情况下，取得了来之不易的成绩。

　　（一）经济增长缓中趋稳。坚持把发展作为始终不变的第一要务，深入开展项目建设和工业稳增长“双百”攻坚行动，推动项目落地投产，促进企业复工复产，培育新的增长点，着力稳定经济增长的基本面，努力增强经济发展后劲。181个在建项目的164个遗留问题得到解决、378个项目开工建设、312个项目储备入库、325个项目建成投产，“六位一体”考核指标全部完成，新开工分类考核重大产业项目94个、完成投资147.26亿元，继续重奖项目建设攻坚战先进县（市、区）、项目建设优秀服务单位，把项目建设攻坚战引向深入。开展系统招商、精准招商、市场招商，“迎老乡、回故乡、建家乡”，推动民企入忻，深化与京津冀区域产业协同发展，签约招商项目总投资2009亿元，到位资金507亿元。继续在“减、免、缓、帮、扶、替”上做文章，大力扶持实体经济。新增直供电企业5户，为企业减负12亿元。开展千名干部入企服务和各类项目受理大起底活动，企业存在问题办结率90%以上。通过千方百计稳增长，攻坚克难保运行，全市地区生产总值增长4.7%、增幅全省排名第三，固定资产投资增长4.3%、增幅全省排名第五，社会消费品零售总额增长7.3%、增幅全省排名第六，一般公共预算收入下降6%、增幅全省排名第九，城镇居民人均可支配收入增长6.5%、增幅全省排名第三，农村居民人均可支配收入增长7.2%、增幅全省排名第二，城镇新增就业3.73万人、登记失业率3.6%，控制在4.2%以内，居民消费价格涨幅0.9%，控制在3%以内。约束性指标全部完成。

　　（二）重点工作有力推进。供给侧结构性改革见到实效。关闭4座煤矿，退出煤炭产能330万吨∕年，完成了省下达的任务，煤矿生产经营好转。商品房销售面积同比增长135.3%，煤、铁、焦、水泥等主要产品库存大幅下降。严厉查处非法集资、高息揽储等违法行为，规范民间借贷行为，切实保障企业资金链安全，企业应急周转资金支持209户企业续贷48.14亿元，“助保贷”累计588户次为企业贷款18.06亿元，繁峙农商行挂牌成立，民营担保公司力天汇通融资公司开业，小额贷款公司经营不断规范。全市经济作物种植面积增长38.8%，全年粮食总产35.34亿斤，为历史上第二个丰收年。

　　产业转型升级步伐加快。晋能保德等低热值煤发电项目扎实推进，全市电力装机突破千万千瓦，金宇超细煅烧高岭土等一批新材料形成规模，阳煤忻通、美新通用等煤机装备制造业集群发展，中科忻能电池项目加快建设，定襄县成功创建全国首家出口法兰锻造产品质量安全示范区，建成省级法兰锻造产品检测检验中心，法兰锻造产业产品延伸到塔筒等部件，以广宇煤电二期为代表的36个企业项目投产；五台山景区行政管理体制改革完成，五台山文旅集团公司成立，综合行政执法试点获批，忻州汇丰文旅投资公司组建，45个旅游项目在政府网站发布招商，芦芽山景区申报世界自然遗产和创建国家5A景区进展顺利，禹王洞晋级国家4A景区，五台县晋察冀军区司令部旧址纪念馆等入选全国红色旅游经典景区名录，忻州被列入第二批国家全域旅游示范区创建单位，全市旅游总收入同比增长15.1%。今年2月雁门关荣膺国家5A级景区，山西第一只市级文化旅游产业投资基金在我市成立，首期规模20亿元。

　　重点领域改革不断深化。开发区体系规划和忻州经济开发区、原平经济技术开发区、各县产业园区规划开始编制，忻州市城市总体规划调整以及县（市）域城镇体系规划和城市（镇）总体规划修编、调整和补充工作同步启动；开发区管理体制理顺，扩区、整合改革积极推进，《开发区（产业园区）管委会领导班子任期制管理办法》《全员岗位聘用制和绩效工资制实施办法》制定印发并启动实施。云河集团商业综合体联合开发项目加快建设，云马焦化破产程序终结，五交化供应站改革重组实施，水务公司法人治理结构不断完善。不动产统一登记制度全面建立。农村土地承包经营权确权工作基本完成。

　　“双创”势头强劲。新登记各类市场主体同比增长31%，忻州市大学生创业园建成投运，培育“小升规”企业22户，建成市中小企业公共服务平台，新认定省级中小企业技术中心6户、高新技术企业4家、省级众创空间5家，实施国家级科技项目1项、省级科技项目12项，全国第五届创新创业大赛山西赛区颁奖典礼暨项目展示活动在我市举办。

　　基础设施持续改善。原平—太原高铁控制性工程进展顺利，原平—大同高铁开工建设，忻州—五台山—北京高铁列入铁总铁路建设“十三五”规划；五盂高速通车，灵河高速完工，神岢高速建设加快，城区30条道路和国道208线忻州市过境改线竣工通车；新建农村公路372.7公里、完善提质146公里；五台山机场连接线项目启动。

　　对外开放不断拓展。以保德、河曲为重点面向陕蒙的西部门户，以忻府、定襄、原平为重点面向太原都市圈的中部门户，以五台、繁峙为重点面向京冀津的东部门户的开放辐射效益得以彰显；五台山机场旅客吞吐量突破11万人，口岸开放纳入国家“十三五”规划；定襄永旺物流园区获批为全市第一家保税仓库和出口监管仓库，列为交通部示范项目。

　　（三）脱贫攻坚精准发力。紧紧把握精准要求，在扶贫对象识别精准、致贫原因查找精准、脱贫帮扶措施精准、政策措施落实精准上下功夫。立足区位存在欠缺、基础设施局限、产业发育不够、天灾人祸影响、个人因素制约和工作存在不足等6种致贫原因，出台了23个专项扶贫行动方案以及45个政策性文件，针对性采取系列脱贫措施。产业扶贫带动贫困劳动力就业14667人，易地扶贫搬迁完成年度投资任务的111.3%，3404名建档立卡贫困人口聘为护林员。90个集中和分布式光伏资产收益扶贫项目有69个已完工，60%的收益用于建档立卡无劳动能力的贫困户分配，18470户贫困农户获得金融扶贫贴息贷款3.9亿元，培训贫困劳动力10690名，对1141名建档立卡贫困户大学生发放补助570.5万元，对8311名中高职学生发放每年每生2000元的助学补助，对40383名贫困大学生办理生源地信用助学贷款2.5亿元，对医疗保障扶贫对象实行“先诊疗后付费”、免除定点医院普通门诊挂号费、大病保险报销起付线由10000元降到5000元，新农合报销比例平均提高2个百分点。全面提高农村低保标准，提前实现“两线合一”。严格扶贫资金使用监管，滞留结转财政扶贫资金清理实现“三个确保”，市县财政扶贫投入分别增长24.28%、54.09%，实现总量、增幅“双增长”。吃透群众需求，激发内生动力，对建档立卡贫困户、贫困边缘户、脱贫户、中等收入户、富裕户分类指导，因户施策，着力让各类群体各得其所，努力建立主动告别贫困、努力向富裕看齐的良好导向。全市脱贫人口8.64万人，出列贫困村576个。

　　（四）致力改善民生事业。在十分困难的情况下认真兑现各项惠民政策。为522名大学生发放一次性求职补贴，为655名大学生购买基层公共服务岗位，托底帮扶安置就业困难人员6769人，失业保险金标准由930元提高到1060元。机关事业单位工作人员增资、县以下机关公务员职务和职级并行、公务用车补贴兑现，全市机关事业单位工资水平全省排名上升。企业退休人员基本养老金人均增加182元，城乡居民基本医疗保险财政人均补助标准提高40元，人均基本公共卫生服务经费财政补助标准提高到45元，城乡居民大病保险年度最高支付限额提高到40万元，县级公立医院药品全部实行零差率销售。为特殊教育学校教师提高津贴50%，生均公用经费提高到6000元/年，农村义务教育学生营养改善工程惠及105468名中小学生，从秋季学年开始市财政投入600余万元支持市直公办中小学校教育教学管理改革。城乡低保、五保、经济困难的高龄与失能老年人补贴按标准提高，困难残疾人生活补贴和重度残疾人护理补贴制度全面实施。社会保障水平稳定提高，机关事业单位、企业基本养老保险制度并轨运行扎实推进，城乡居民基本医疗保险完成初步整合，养老服务体系进一步完善，新增养老床位数1500张。教育事业不断发展，新建忻州高级中学、长征小学、实验幼儿园等竣工，改扩建农村幼儿园21所，代县、原平市、河曲县、五寨县“创建全国义务教育发展基本均衡县”顺利通过省级督导评估和国家认定，县级政府推进义务教育均衡发展工作得分全省排名第一。县级公立医院综合改革全覆盖，医用设备检查检验费降幅超过10%，创建国家级、省级群众满意的乡镇卫生院37所。县级图书馆、文化馆和乡镇文化站全部免费开放，市“五馆一院”建设加快，图书馆、博物馆主体封顶。定襄等6个县建成城市数字影院，一批世界遗产点和国家、省级重点文物保护修缮工程完工。全民健身活动广泛开展，竞技体育水平全面提高，省十五届运动会摘金任务超额完成，全国中国式摔跤冠军赛暨挠羊赛在忻州举办。忻州市第七次蝉联“全国双拥模范城”桂冠。

　　（五）着力改善忻州形象。以列入全国10个开展商务综合行政执法体制改革试点城市之一为契机，市县两级全部建立健全商务综合执法机构，全面开展对商务流通市场的监管，商务综合行政执法体制改革试点工作获评全国第二。认真落实“党政同责、一岗双责、齐抓共管、失职追责”要求，不断深化安全生产“4438”工作机制，重点加强安全风险预控管理和应急救援体系，夯实乡村监管基础，切实做好安全生产各项工作，全年事故起数同比下降7%，安全生产考核排名全省第一，忻州市入选中国城市竞争力研究会权威发布的2016中国最安全城市排行榜。实施“五城联创”，坚持全民创卫、依法创卫、集约创卫、和谐创卫，忻州城区创建国家卫生城市通过暗访，今年通过了技术评估；忻州市荣获全省城乡爱国卫生清洁运动综合考核设区市第一名，原平市荣获全省城乡爱国卫生清洁运动综合考核县级市第一名。城市综合信用指数全省排名第二。生态环保全面加强，忻州城区创建省级园林城市通过验收并授牌，全市营造林71.48万亩，静乐县开展政府购买式造林试点；深入开展“铁腕治污行动”，严厉打击各类环境违法行为，加大以雾霾为重点的环境污染治理力度，设区市城市空气质量优良天数比例完成年度任务。

　　（六）自身建设明显加强。出台《忻州市法治政府建设实施意见（2015—2020年》，确保政府工作在法律和政策范围内活动。制定实施《忻州市全面加强政府自身建设2016年行动计划》，努力建设“六型”政府，切实把市政府“执政为民、敢为人先、群策群力、滴水石穿”的政府精神和“约法五章”落到实处。认真办理人大代表建议和政协提案，全年办理省人大、省政协建议提案4件、市人大代表建议287件、市政协委员提案330件。持续提升“忻州随手拍”问政服务平台、“忻州发布”政务微博运行水平，忻州随手拍荣获全国“互联网＋政务传播创新奖”，被评为“移动政务创新十佳案例”。主动回应、积极引导、认真处理涉及城镇化发展、民生民情、安全隐患和事故、群众网络留言等网络舆情，实现网络问政常态化，省长信箱转办、督办的78件事项办结76件，市长信箱反映的65件事项办结60件，全年重要舆情同比减少150条。深化“放管服效”改革，继续取消、下放、清理规范一批行政审批和中介服务事项，加强事中事后监管，建立健全权责清单动态管理和行政职权运行监督管理制度，市本级权力清单由2689项调整为2679项，行政许可事项由201项调整为196项，各县（市、区）“两单两图”均在政府门户网站公布。实施“互联网+政务服务”，市县两级政务服务平台和市级公共资源交易平台基本建成并投入运行，行政审批“两集中、两到位”全面推行，在全省首家实现“13710”电子督办系统市县乡三级政府全覆盖。

　　扎实开展“两学一做”学习教育，持续加强政府系统党风廉政建设和作风建设，切实增强政治意识、大局意识、核心意识、看齐意识，自觉理政、谋政、勤政、善政、廉政，勇于担责、担诺、担急、担难、担险、担远，时刻把纪律和规矩挺在前面，严格执行中央八项规定和国务院“约法三章”精神，持续狠刹“四风”，厉行勤俭节约，“三公”经费持续下降，着力树立为民、务实、高效、担当的政府形象。

　　这些成绩的取得，是省委、省政府亲切关怀、重视支持的结果，是市委统揽全局、正确领导的结果，是市人大、市政协大力支持、有效监督的结果，是全市广大干部群众同心同德、攻坚克难的结果。在此，我代表市人民政府，向全市人民，向各民主党派、工商联和无党派人士，向各位人大代表、政协委员，向驻忻人民解放军、武警官兵、公安民警和驻忻单位，向所有关心支持忻州改革发展进步的同志们、朋友们，表示崇高的敬意和衷心的感谢！

　　我们清醒地知道，当前我市还存在许多深层次的问题，特别是传统产业转型升级尚处于破题阶段，新兴产业发展缓慢，核心竞争力远未形成，重点领域改革仍然滞后，尤其是还远不能适应新常态的要求。归结于一点，就是在本轮经济的深度调整中，如何化危为机，发挥后发优势，实现转型跨越仍在探索之中。具体表现在：市场环境短期内难以根本好转，经济尚未进入合理增长区间，经济增长面临很大的不确定性，仍没有走出困难时期。经济总量小，产业层次低，经济结构性矛盾突出；实体经济面临不少困难，企业亏损面较大，投资积极性不高，缺乏大的项目支撑，经济增长的内生动力不足；财政收入仍为负增长，收支矛盾突出，保民生、保运转、保工资压力还在继续增大；高校毕业生和转移农村劳动力总量高于去年，安全生产、社会治安、公共安全等领域存在风险隐患，社会稳定压力加大；城乡发展不平衡，民生社会事业欠账多，公共服务与人民群众日益增长的物质文化需求不相适应，补短板任务艰巨；资源环境约束加剧，环境保护基础薄弱，大气、水、土壤污染问题突出，尤其是雾霾问题日益成为人民群众关注的焦点；上级、群众对政府工作的要求越来越严、越来越高，不少干部在思维观念、工作方式、纪律作风、自身能力等方面还不适应事业发展和工作需要，等等。解决这些问题和困难关键在于我们自己。我们要立足现实，着眼未来，一切从自己身上找原因，一切立足于自己努力解决问题，靠苦功夫、笨办法，做艰苦细致的工作和坚持不懈的努力，始终保持力争上游、百舸争流的竞进态势，奋力开启忻州发挥后发优势，实现奋勇争先，全面振兴崛起的新征程！

　　二、2017年工作安排

　　2017年，是推进供给侧结构性改革和转型综改的深化之年，是走出经济困难局面的攻坚之年。做好今年的政府工作，要深入贯彻习近平总书记系列重要讲话精神和治国理政新理念新思想新战略，统筹推进“五位一体”总体布局，协调推进“四个全面”战略布局，认真贯彻中央经济工作会议精神和省第十一次党代会、市第四次党代会总体部署，坚持“一个指引、两手硬”重大思路和要求，坚持新发展理念，坚持稳中求进工作总基调，坚持脱贫攻坚统揽经济社会发展全局，以深化供给侧结构性改革与深化转型综改试验区建设为主线，以提高发展质量和效益为中心，全面实施“1661”发展战略，全力做好稳增长、促改革、调结构、惠民生、防风险各项工作，促进经济稳步向好和社会和谐稳定，加快全面脱贫、全面小康进程，以优异成绩迎接党的十九大胜利召开。

　　主要预期指标是：GDP增长5.5%左右，全社会固定资产投资增长5%，社会消费品零售总额增长7%左右，一般公共预算收入下降3.7%，城乡居民人均可支配收入分别增长6.5%左右和6.5%以上，居民消费价格涨幅控制在3%左右，城镇新增就业2.9万人，城镇登记失业率控制在4.2%以内。

　　约束性指标是：资源节约方面，万元GDP能耗、万元GDP二氧化碳排放量、万元GDP用水量，按省下达任务执行。环境质量方面，PM2.5浓度下降3.5%、空气质量优良天数比例为72.4%（265天）、达到或好于Ⅲ类水体比例为78.57%、劣V类水体比例为0，化学需氧量、氨氮、二氧化硫、氮氧化物排放量等其他4项约束性指标，按省下达任务执行。民生改善方面，农村贫困人口脱贫人数11.9万人，城镇棚户区住房改造3830套。

　　为突出转型导向，今年设定转型指标体系。即传统产业占工业的比重下降1.5个百分点，煤炭产业占工业的比重下降2.5个百分点，战略性新兴产业占工业的比重提高1个百分点，服务业占全市经济的比重稳定在50%左右。

　　重点做好以下10个方面的工作：

　　（一）切实发挥转型综改的牵引作用，着力推动产业结构优化升级。

　　改造提升传统产业。继续推进新型综合能源基地建设，强化煤炭生产经营监管，推动矿井建设向集约高效型转变，推进保德王家岭500万吨/年煤矿及配套洗选项目尽快投产，确保煤炭经济平稳运行。推动煤电联营，加快华润宁武、山煤河曲、晋能保德等3个低热值煤电厂等项目进度，配合晋北—江苏特高压通道建设，加快80万千瓦风电及15万千瓦光伏发电项目建设。加快500千伏华润宁武—朔州送出工程建设，做好晋能保德、山煤河曲500千伏送出工程协调保障工作，争取500千伏繁峙变电站等外送通道项目，开工220千伏繁峙滹源等8项工程，投产110千伏忻府部落等6项主网基建工程，为全市煤电基地建设、新能源送出奠定基础。加大传统产业技术改造，加快传统产业与新技术、新工艺、新模式相互嫁接，推进保德彬凯、忻州奇石、忻州铁牛等3个60万吨水泥粉磨站技改项目，完成鑫盛铁矿等15个企业升级改造，提升铁精粉等产品竞争力。推动同德铝业理顺股权结构、尽快复工，力争2018年底建成投产。推进岢岚道生鑫宇煤化工、禹王煤化工做大做强。深入开展消费品工业“增品种、提品质、创品牌”专项行动，推动食醋、白酒、乳制品、陶瓷、工艺品等产业提档升级。

　　培育壮大战略性新兴产业。大力发展数字经济。成立大数据产业局，认真落实与浪潮集团战略合作协议，共同推动信息产业创新发展；全力引进北大方正，抓好从传感器、读取器、传输设备到处理设施的数据产业链；推动移动、联通、电信、广电的数据平台项目尽可能在忻州落地；抓好忻州政务云、智慧城市、代县暖洋洋铁矿采选智能控制系统、五台山国际沙棘健康养生旅游基地、静乐小杂粮电子商务、管家营法兰ERP海宝软件等大数据发展应用项目。大力发展装备制造业。推动繁峙由铁矿粉向铸件、铸件向部件延伸；抓好定襄风电塔筒，推动低端法兰向高端法兰转型，推进已研发成功的双轴智能车床、节能电机、人造金刚石压机等整机进入中试；加强阳煤忻通与德国布朗公司合作，提升煤机装备制造水平；抓好华夏新能源汽车铝合金车身研制、双环重工风电关键结构件、宝龙达锻造5000吨高级双相不锈钢阀门零配件、晨辉锻造机械手、一辰暖通水动能潆射供热机组项目建设，推进德奥电梯立体车库项目投产运营。大力发展新兴潜力产业。抓好金宇煅烧高岭土三期项目、蓝天锅炉、中恒制药甘净养肝产品、培森生物800万人份新型疫苗、云中制药、嘉兴制药等项目，推动中石油保德煤层气深度开发、利国磁性材料年产20万吨高性能取向硅钢等一批项目。

　　大力发展现代服务业。发挥“互联网+”在服务业中的作用，加快市级“一网两平台”建设，大力发展农村电商。加快发展和积极引进公证、律师、会计及咨询、招标、拍卖等生产服务业和中介服务组织，建成国家级法兰检验中心。加快发展现代物流产业，永旺物流园区保税仓库和出口监管仓库投入使用。大力发展康养产业，突出打造忻府区顿村、奇村以及五台山、砂河、东寨等集旅游、度假、养老、医疗为一体的康养产业集群。

　　加快把文化旅游产业打造成战略性支柱产业。围绕“五个一批”“八个加强”“六字要诀”，突出三个理顺、三个加强，加快“三个集散地”建设，加快推进全域旅游示范区创建工作。加快景区的分类和景区管理体制、经营体制改革，对适合组建经营主体的，推进所有权、管理权和经营权分离，以经营权面向市场招标，确定经营主体；对不宜组建经营主体的景区，进一步完善管理，确保五台山景区、芦芽山景区、雁门关景区等省市确定的首批34个景区（景点）上半年如期完成体制机制改革创新任务。抓好五台山风景区整治整改、经营体制改革、科考线路开发、农村客栈建设、综合行政执法试点，实现严格保护、科学开发、精心利用，带动全域旅游发展。运作好文化旅游产业发展基金，发挥好“五台山号”旅游列车的推介作用和忻州市旅游行业协会行业的自律作用。加快芦芽山申遗工作，加快芦芽山、阎锡山故居创建国家5A级旅游景区，以及代县古城等8个景区创建国家4A级旅游景区工作。加快打造一批旅游精品线路。开通由旅游集散中心通往五台山、芦芽山、雁门关等主要景区（点）的旅游直通车，实现所有A级景区手机信号、“WIFI”和电子监控全覆盖、无盲区。

　　加快推进“336”综改布局。坚持不懈建设以保德、河曲为重点的西部门户，以忻府、定襄、原平为重点的中部门户，以五台、繁峙为重点的东部门户；坚持不懈打造东部五台山，中部奇村、顿村温泉度假区，西部芦芽山“三个集散地”；坚持不懈发展河曲、保德煤电铝化板块，神池、五寨、岢岚、偏关现代农业板块，宁武、静乐煤电化板块，忻府、定襄、原平多元产业板块，繁峙、代县矿业冶炼板块，五台旅游板块等6大特色区域经济板块。

　　（二）大力实施创新驱动战略，提升企业的核心竞争力。

　　强化理念创新。当前我们缺的东西很多，短板很多，但最大的短板、最缺乏的还是干部队伍对未来的信心以及新的发展理念和有力的执行力。要树立新型的开放观。自觉运用“地球村”的眼光和方式来认识、思考问题，着眼一流、体现差异、守住底线，真正打开心门、打开山门、打开城门。要树立新型的市场观。把市场作为反映需求紧缺程度的晴雨表和决定企业生死存亡的“无形的手”。要树立新型的资源观。跳出煤炭资源依赖，真正把文化、生态、市场、环境、效率、法治、诚信等发展要素作为宝贵资源，把企业家作为忻州最稀缺的资源，把人才作为最重要的第一资源。要树立新型的竞争观。敢于否定自己、不断超越自己，与时代赛跑，与未来竞争。要树立新型的人才观。人人都是人才，人人都可以成才，做出贡献的就是人才。要树立新型的发展观。把深化供给侧结构性改革与深化综改试验区建设作为经济工作的主线，由“找凤凰”转为“种梧桐树引凤凰”，促进新发展理念的落地生根，更加注重园区发展、板块发展、集聚发展，更加注重发展新兴产业，更加注重创新驱动，更加注重开放体系、中介服务、岸港网建设。

　　强化经营创新。一切以满足消费需求为标准，不断以新的经营方式、新的经营技术、新的经营手段创造出不同形式、不同风格、不同组合的商品形态。努力从研发设计环节分解形成独立的研发设计企业，从采购和销售环节分解形成第三方物流企业，从售后服务环节分解形成专业的售后代理公司，从人力资源管理环节分解形成专业咨询公司，从财务管理环节分解形成会计师事务所，从法律事务环节独立形成律师事务所，不断创新管理模式、经营模式、创业模式、营销模式、商业模式和营利模式。

　　强化科技创新。市级设立6000万元企业技术改造扶持资金，发挥财政资金“四两拨千斤”的引导作用。切实强化企业创新主体地位，打通科技和经济社会发展的通道。实施煤电一体化、锻造精加工等科技重大项目，支持阳煤忻通装备制造、中科忻能电动汽车、培森生物制药、管家营法兰企业技术改造等科技攻关，争取利国磁性材料等3家企业通过高新技术企业认定，新认定省中小企业技术中心2个以上；进一步完善科学技术奖励办法，奖项设置更加切合实际，奖金数额更能体现价值追求；用好国家级技术转移示范机构平台，全市技术交易额3亿元以上，一批专利技术和科技成果真正得到转化，建成2个省级农村“星创天地”；保持有效发明专利拥量增长势头，建成公开统一的科技管理平台和科技管理信息系统。

　　强化金融创新。综合运用财政、资本、市场手段，着力加快进度、打造平台、依法办事、防范风险，为保发展、保民生、保基本提供可靠的融资保障。做实企业信用基础，不断提升企业信用等级，加快培育合格主体；巩固“助保贷”“助羊贷”“过桥资金”成效，鼓励金融机构采用展期、转贷、降息等方式，创新融资手段，按照风险可控原则，缩短审批环节、扩大抵押物范围、灵活利率定价，提供全方位综合金融服务，持续提升服务实体经济能力，千方百计保障企业资金链安全。继续开展政银企项目对接活动，全力保障重点工程、重大项目的资金需求。加大对“三农”、脱贫攻坚、中小微企业等薄弱环节的金融支持。继续推进农信社改制，加快原平、保德联社组建农商银行步伐，确保年内挂牌开业。积极推动企业进入资本市场，优化上市（挂牌）资源库，争取早日挂牌一批，形成示范带动效应。完善打击非法集资长效机制，严格防控不良贷款大幅反弹，规范有序发展小额贷款公司，防范和处置各类金融风险，不断优化金融生态环境。

　　（三）深化供给侧结构性改革，构建多元现代产业体系。

　　坚定去产能。重点抓好煤炭去产能，更好运用市场化、法治化方式，严格执行环保、能耗、质量、安全等相关法律法规和标准，坚持淘汰落后产能与发展先进产能相结合，实施减量置换、减量重组，坚定去产能、稳产量、保安全、增效益，加快煤炭绿色清洁高效利用。

　　着力去库存。支持企业搞好产销衔接，降低工业产成品库存。继续做好“忻材忻用”工作，加大市重点工程使用市内钢材、水泥、用工的统计、督查、协调和考核，凡涉及政府主导的基础设施和公益性项目、使用财政资金采购重大装备和产品的项目，在材料、设备招标采购中，同等条件下要优先选用本市产品，努力提高我市地方产品的使用率。加大棚户区改造和城中村改造货币化安置力度，全面打通商品房、保障房和安置房转换通道，结合易地扶贫搬迁等政策，采取多种措施，两年内完成房地产去库存任务。

　　稳步去杠杆。防控资产泡沫和金融脱实向虚。用好国家对贫困县企业IPO和挂牌“新三板”“即报即审、审过即发”等政策，建立贫困县上市企业引导基金，引导更多的企业在忻州落地，推动企业上市有新突破，融资总量突破5亿元；做好金融助推脱贫攻坚工作，不断提升11个国定贫困县贷款增加额全市占比，建档立卡贫困户贷款覆盖率高于全省平均水平。

　　多措降成本。坚持“四堵墙内的事企业办，四堵墙外的事政府办”，进一步落实国家、省《降低实体经济企业成本工作方案》和省政府“减负60条”“工业20条”以及市“减负48条”“工业26”条等各项减税降费政策，继续在“减、免、缓、帮、扶、替”上做文章，合理降低企业人工成本、用能用地成本和物流成本，支持实体经济发展。鼓励企业挖潜创新，苦练内功，大力开展精细化管理，降低生产成本，压缩期间费用，在提高劳动生产率和利润率上下功夫。

　　（四）不断引深项目建设攻坚战，切实增强发展后劲。

　　抓好重点投资项目。实施省市两级重点工程项目74项，总投资991.08亿元，年度计划投资179.96亿元。其中：省级重点工程项目24项，总投资351.81亿元，年度计划投资37.1亿元；产业转型重点工程项目14项，总投资272.3亿元，计划完成投资24.6亿元；基础设施重点工程项目10项，总投资79.5亿元，计划完成投资12.5亿元。市级重点工程项目50项，总投资639.27亿元，年度计划投资142.86亿元。其中：产业转型重点工程项目15项，总投资310.35亿元，年度计划投资53.73亿元；基础设施重点工程项目35项，总投资328.92亿元，年度计划投资89.13亿元。

　　完善项目考评机制。继续实行重大产业项目分类考核，抓好14个县（市、区）、忻州经济开发区、五台山景区每家9个共计144个的重大产业项目建设。坚持“八位一体”推进机制，完善重大产业项目考核办法，突出项目落地、投资、建成投产三个环节，从项目筛选到项目建设全程，落实政府推进主体、部门服务主体、企业实施主体“三个主体责任”，确保重大产业项目始终在强有力的组织领导下顺利推进。继续开展全市项目观摩和项目考核评比活动，实行重大项目奖励制度。

　　提升招商引资水平。围绕产业链条、产业集群、产业目录，以开发区、产业聚集区为依托，发挥各类商会的桥梁作用，积极融入京津冀和环渤海经济圈，承接发达地区产业转移；实施专业招商、以商招商、会展招商、网络招商、委托招商，在招引世界和国内500强企业上实现突破，力争全年招商引资项目到位资金500亿元以上。改进和完善招商引资考核体系，加大合同履约率、资金到位率、项目开工率考核权重。启动实施“忻商忻才回乡创业创新”工程，鼓励和吸引忻商忻才回流回乡、创新创业、投资发展。

　　（五）坚决打赢脱贫攻坚战，认真做好“三农”工作。

　　强力推进脱贫攻坚。坚持精准扶贫、精准脱贫基本方略。深入开展特色产业扶贫。完善产业项目与贫困户利益联结机制，推进贫困村“一村一品一主体”全覆盖，带动6万以上贫困群众增收；深入推进转移就业扶贫，开展以“忻州月嫂”为主打品牌的技能培训；稳步实施光伏扶贫，惠及89个建档立卡贫困村、7500个建档立卡贫困户；深入推进生态补偿扶贫，退耕还林奖补优先保证贫困户，一般公益林、国家级公益林管护任务的60%安排给贫困户，实施干果经济林提质增效8万亩；大力推进旅游扶贫，实施好26个旅游扶贫项目，建设休闲农业，搞好农旅互动；认真落实省支持忻州脱贫攻坚的五项政策，确保取得实效。扎实推进易地扶贫搬迁。精准落实搬迁对象，确保去年项目全部竣工、2万人入住，今年完成2万贫困人口搬迁、5500人同步搬迁的新开工任务；搞好50户以下贫困村整体搬迁工作；采取集中供养、日间照料中心、低保+医保+养老等模式，因地制宜解决好老年人搬迁安置问题。着力改善贫困村基础设施、公共设施、村容村貌，加强搬迁后的贫困村宅基地管理。进一步加大教育、民政、健康、社会保障等专项扶贫政策到村到户到人到位落实力度。健全完善家庭经济困难学生资助体系，减轻贫困家庭教育支出负担；完善农村低保制度与扶贫开发政策有效衔接，逐步提高贫困地区基本养老保障水平；构建新农合、大病保险、健康扶贫补充保险为主，医疗救助、临时救助、慈善救助为补充的健康扶贫兜底体系；落实特殊群体补贴政策，实施残疾人精准康复服务行动；完善扶贫小额信贷政策体系，推广政银保企农“五位一体”模式，信贷规模达到9.2亿元；以财政资金和金融资金折股量化为支撑，在提升贫困户财产性收入上实现突破；加大财政投入和资金整合力度，确保财政扶贫资金投入总量和增幅继续保持“双增长”；推进扶贫开发资源整合，最大限度将惠民政策、项目和工程向贫困县、贫困村、贫困人口倾斜，确保今年保德县脱贫摘帽，642个贫困村出列、11.9万贫困人口脱贫。

　　加快农业产业化。坚持以工业化理念，走产业化路子，以“一县一业一园区”为抓手，加快北方农牧交错带国家级示范区建设，着力打造知名品牌，壮大龙头企业，培育营销主体，建设产业基地，创新经营模式，力促农业加快转型，促进一二三产业融合发展，推动由农业资源大市向现代农业强市的跨越。进一步振兴忻州杂粮产业，组织实施“杂粮之都十大创建工程”，启动农产品电子商务物流园区项目建设。提升畜牧产业水平，新建、完善和提升30个现代化养殖园区、200个家庭畜牧养殖场和500个规模养殖户。新增认证无公害绿色有机和地标农产品50个以上、认定“三品”原料生产基地10万亩以上。发展“农超对接”“农企对接”“农店对接”，力争县级农村电商运营中心达到10个以上、村级电商服务网点达到1000个以上。培育专业种养大户，发展专业家庭农场，壮大农民专业合作社，提升农业产业化、组织化水平。

　　大力发展功能农业。加强农业供给侧结构性改革，提高农业供给体系质量和效率。坚持农产品营养化、功能化发展方向，面向中高端市场，推进药食同源产品开发，大力发展藜麦、玛卡、黑枸杞等高附加值农产品，加快忻州特色农产品药食化、功能化、主食化、副食化。突出抓好晋北原料药基地建设，依托东西两大山区生态资源优势，建设东部五台山、恒山和西部管涔山、云中山两大中药材产业基地，发展5大道地中药材及食药同源产业、培育10大龙头企业、100个规模合作社，全市种植面积达到30万亩，为生物医药发展提供原料保障。

　　加强农业基础建设。落实各项强农惠农政策，以提高耕地地力、建设标准农田为重点，完成高标准农田建设23.4万亩，新增灌溉面积17万亩，建成千亩以上地膜旱作丰产方100个以上。建立完善服务体系，培育多种形式的农业经营性服务组织。引导完善农业保险政策，拓宽农业保险范围，加大保费补贴力度，构建方便快捷的保险服务网络。

　　改善农村人居环境。开展农村建筑特色风貌整治，实施农户庭院美化、农村街巷整治、村庄净化绿化“三大工程”，加大改气、改水、改厕力度，推进美丽宜居乡村建设。完成采煤沉陷区治理搬迁958户2656人、农村地质灾害治理387户、城中村改造1709户、农村危房改造7014户。抓好美丽宜居示范村提档升级和重点传统村落保护及开发利用。

　　（六）不断提升竞争软实力，打造投资洼地、创业乐土。

　　持续改善基础设施。坚定不移把城镇化作为主攻方向，抓好大同至原平高速铁路、神池至岢岚高速公路建设，加快推进基础设施内联外通。推进“小水网”配套项目建设，争取部分输水和调蓄引黄水项目开工建设。实施忻州城区南部改造、中部提升、北部开发。市区实施好44条市政道路，以及九龙岗森林公园、云中河景区二期、体育公园等工程建设，提升、巩固“创园”“创卫”成果。完成秀容书院修复、古城和历史文化街区保护。建设忻州—定襄、忻州—原平城际快速通道，建成机场高速连接线。实施农村旅游公路规划项目建设，规划建设108、208国道过境汽运物流服务中心。持续推进“五城联创”，加快提升中心城区的核心竞争力和知名度、美誉度。优化空间布局和功能，提高城市规划、建设、管理水平，深化特色小城镇建设。组织实施好政府购买服务项目，努力为广大人民群众提供优质高效的公共服务。

　　优化生产要素供给。按照统一市场、公平竞争的标准，采取综合措施，优化土地、资金、技术、劳动力等生产要素配置，促进实体经济加快发展。做好涉及市场准入、产业发展、招商引资、政府采购、行为规范、资质标准等政策措施的审查工作，保障所有市场主体平等使用生产要素，同等受到法律保护。培育和健全完善生产生活服务体系，对应中介服务组织目录建立专家库，为在忻投资企业提供便捷服务。

　　创造良好的社会环境。牢固树立“企业家是最稀缺资源”的理念，尊重企业家的创造，真心实意为企业服务，不折不扣地解决企业遇到的困难和问题。深入开展企业周边地区和突出治安问题专项整治，为项目建设和企业生产经营保驾护航。

　　维护良好的市场秩序。坚持放管并重，实行宽进严管，着力加强对重点区域、重点领域、重点环节和重点产品的监管，切实解决食品药品、生态环境、安全生产、金融服务、网络信息、电子商务、房地产等领域扰乱市场秩序、侵害消费者合法权益的问题，营造公开、公平、公正的竞争环境，激发市场内在活力，保障经济社会持续健康发展。

　　加强诚信忻州建设。建立和完善诚信体系，加快建立统一的社会信用平台，运用市场主体信用信息系统，推进“一处失信，处处受限”的信用约束机制形成，全面深化诚信县、乡、村、单位、企业创建活动。大力倡导契约精神，不断强化信用约束，着力解决不诚不信的问题，切实增强全社会的诚信意识和信用观念，在全市上下形成人人讲诚信、个个守信用的浓厚氛围。

　　大力弘扬先进文化。牢牢把握意识形态工作领导权、话语权，弘扬改革创新的时代精神、转型综改、振兴崛起的发展主旋律，提振精气神、汇聚正能量。注重发挥社会主义先进文化、优秀传统文化、红色文化资政育人作用，讲好忻州故事，传播忻州声音，展示忻州形象。实施“文明守望工程”，动员社会力量参与文物保护利用，挖掘传统文化，把文化资源优势转化成发展优势和竞争优势。加强公共文化基础建设，促进文化与科技、信息、旅游、金融等产业融合发展。

　　不断扩大对外开放。加快推动五台山机场航空口岸开放。积极参与山西品牌丝路行活动，推动我市特色企业、特色产品走出去，擦亮忻州品牌。

　　（七）深化重点领域改革，不断释放政策红利和发展活力。

　　加快开发区改革创新发展。抢抓全省推动开发区改革创新发展的历史机遇，推动开发区体制机制“二次创新创业”。按照《忻州市开发区改革创新发展实施方案》，推行“五规合一”，实现“一张图”工程，科学修编全市开发区总体发展规划，推进开发区整合、改制、扩区、调规。支持忻州经济开发区扩区整合，建设国家级开发区；做大做强原平经济技术开发区；推动定襄、繁峙、宁武、河曲、保德、岢岚等6县建设经济技术开发区，代县、偏关两县建设文化生态旅游开发区，五台、静乐、五寨、神池等4个县建设现代农业产业开发区。围绕煤炭清洁高效利用、新型综合能源、新型电子产业、煤化工、装备制造、绿色农产品加工、文化旅游、现代物流和电子商务9大产业，科学确立开发区首位产业，引进实施主导产业。全面实施“三化三制”改革，理顺开发区管理体制、运行机制和各类利益关系，建立专业化、市场化的管理运行机制，实行领导班子任期制、全员岗位聘任制和绩效工资制，真正激发开发区内部活力，把开发区建设成新兴产业“集聚区”、先行先试“试验田”、对外开放“桥头堡”、创新驱动“主引擎”、全面小康“加速器”。

　　深化企业改革。按照小转大、大转强、强转股、股上市的目标，深化优化国企改革。坚持一企一策，通过做强一批、整合一批、重组一批、转型一批、股改一批、注销一批、破产一批、划转一批，分类推进市属35户国有企业改革。采取公司分立、推动人资分离、妥善安置职工、推动企业转型、创新经营模式“五步工作法”，积极探索一批市属国有企业改革的典型，做到国有资产不流失、人员生活有保障。坚持国企国资改革方向，突出市场化、法治化取向，强化管理，加强合作，推动国有企业瘦身健体、固本培元、提质增效、转型升级。在盘活资产及土地和企业转型发展上做文章，解决好停产、半停产企业职工的生活出路问题。全面实施中小微企业规范化改制三年行动计划，引导规模以上重点企业完善法人治理结构，建立研发、管理、营销团队，加快向现代企业的跨越。着力推动“有”转“优”进一步调优存量，推动“无”转“有”进一步做优增量，推动“农”转“工”进一步做优农业，推动“散”转“聚”进一步做优园区，推动“短”转“长”进一步做优链条，靠延伸、靠衍生、靠提升、靠扩张，促进企业转型升级，不断提高经济增长的质量和效益。

　　深化电力体制改革。探索局域电网运营新模式，扩大直供电规模和范围，提高自备电装机比重，有效降低企业用电成本。积极实施火电机组节能改造，探索电力消纳新出路。

　　深化农村产权制度改革。完善农村土地所有权、承包权、经营权分置机制，总结定襄、岢岚2县农村土地承包经营权抵押贷款试点工作经验，积极稳妥扩大试点范围。推进农村产权流转交易市场建设，建立农村产权信息管理平台，做好农村集体产权确权到户试点工作。加快推进国有林场改革，继续深化集体林权制度改革。深入推进供销合作社综合改革。

　　深化行政审批制度改革。继续做好上级政府取消、下放、调整行政审批项目的承接工作，进一步取消、下放行政审批项目，对照梳理调整市本级行政审批项目目录，推动政府职能转变，激发市场活力。

　　深化投融资体制改革。落实企业投资自主权，实行企业投资项目管理负面清单、权力清单、责任清单制度。发挥好政府投资的引导和带动作用，完善PPP有效推进机制，对传统基础设施、公益性等重点领域的投资项目实行政府购买服务。

　　深化综合行政执法体制改革。科学界定执法监管职责，有效整合相同行业执法体制，重点在质量监管、市场监管、药品监管、卫生计生、文化旅游、商务流通、城乡建设、城市管理等8个领域全面推开综合行政执法体制改革。探索建立行政执法全过程记录、重大行政执法决定法制审查等制度。

　　（八）大力强化人才支撑，不断促进创业创新。

　　开展“教育振兴年”行动。坚持科教兴市、人才强市，把今年确定为忻州的“教育振兴年”。启运忻州高级中学、长征小学、实验幼儿园；新建第十四中学、雁门小学、龙岗小学、和平小学等；改造忻州十二中、第二实验小学、实验小学，提升忻州职业技术学院，新建忻州高级技工学校，筹建忻州农业职业技术学院、旅游职业学院，形成从幼儿园、小学到初中、高中、大学，从农业、工业到服务业较为完整的教育体系，不断夯实人才强市基础。切实加大教育投入，认真落实教师待遇，切实加强校长和教师队伍建设，不断提升教育质量。

　　以“双创”带动就业。多层次培育“双创”主体，鼓励以创业带动就业。继续安排3000万元全民创业专项扶持资金和3000万元中小企业发展专项资金，发挥大学生创业园的孵化作用，推动大众创业、万众创新。深化创业型城市创建工作，组织创业项目洽谈对接，提高创业成功率。创建“双创”示范基地，支持新建基地型、旧企业闲置厂房改造型、城市楼宇型等小企业创业基地发展，让各类创业者“拎包创业”。培育认证一批众创空间，力争达到省级以上标准。支持个转企、小升规、规改股、股上市，推进创新型中小企业梯次发展、龙头企业做大做强。加强双创政策支持，腾出资金保基本、保战略、保就业，提高基本公共服务保障水平。

　　强化知识价值导向。实施以增加知识价值为导向的分配政策，积极引进、培养、用好各类科技人才、企业经营管理人才、高技能人才和农村实用人才。改革事业单位编制和人事管理模式，全面落实用人主体自主权，改革人才评价制度，打通政企、事企人才双向流动通道，取消机关事业单位进人控编卡、计划卡，构建全新的人才自主流动机制。取消附着在职称评聘上的非专业条件和限制性要求，实行评聘分离制度，给人才松绑。实施“一园十站双百聚才计划”，推进“百人计划”“千人计划”扩容增量，统筹推进专家工作站和大学生创业园建设。建立忻州籍在外专家人才目录库，完善联系服务机制，深入开展迎老乡、回故乡、建家乡活动。围绕重大项目、新兴产业，举办新闻发布会、招才引智会，多形式、多渠道、多途径征集人才需求目录，加大知名人士、院士的柔性引才力度。加强对市管优秀专家发挥作用的考核，完善专家服务基层积分卡制度，畅通专家人才进出渠道，激发人才活力、潜力和创造力。把开发区作为人才聚集和产业发展的高地，围绕重点产业和新兴产业，完善优惠政策，引进国内外高端、紧缺人才，做到报酬与待遇相匹配、价值与贡献相统一；解决好人才配偶就业、子女就读、住房保障等后顾之忧，用政策吸引人才、以平台凝聚人才、靠服务留住人才，引进一个人才，带动一批项目，聚集一支团队，发展一方产业。

　　充分发挥乡土人才作用。把乡土人才发展纳入人才整体开发规划和经济社会发展总体规划，创新乡土人才选拔、培养、管理、使用机制，充分挖掘乡土人才在知识、技术、资源等方面的优势。改革评价方式，为乡土人才创业提供支持、营造环境，激励他们扎根基层，充分发挥“培训一批能人、带动一方百姓、搞活一片经济”的人才效应。注重向基层一线、脱贫攻坚主战场倾斜，以返乡青年农民、种养加能手、专业大户、科技示范户、农业产业化龙头企业科技人才为培养重点，定期举办农民急需的、与市场对接的技术培训、政策咨询，组织开展“土专家”“田秀才”现身说法，促进成果转化，让这些乡土人才在一线发挥作用,发挥好乡土人才在经济社会发展特别是推进脱贫攻坚中的独特作用。

　　强化企业市场主体地位，保护企业家精神。聚焦产业、企业、企业家，拓展干部入企服务实效，建立完善服务企业长效机制，在市经信委设立服务企业办公室，继续在为企业“减免缓帮扶替”上下功夫，最大程度保护企业家的积极性、主动性和创造性。构建亲清政商关系，为企业发展和企业家创业创造好的发展环境。着力提升企业家素质，组织重点企业家到发达地区定向、定点培训，解放思想，开阔眼界，培育一批现代化的企业家；加强企业后备人才培养，把民营企业二代人才培育成富有改革创新精神、敏锐市场眼光的新一代企业家，努力在激烈的市场竞争中赢得主动，占领制高点。

　　（九）坚持以人民为中心，不断保障和改善民生。

　　着力稳定和扩大就业。加快发展服务业等劳动密集型产业，扶持中小微企业和民营企业发展，开发更多就业岗位。认真组织开展“就业援助月”“春风行动”“公共就业服务进校园”等就业专项活动，搭建就业供需平台。实施政府购买基层公共服务岗位、“三支一扶”、就业见习等基层服务项目，吸纳高校毕业生就业。发挥就业专项资金和失业保险基金作用，引导和支持企业采取多种办法稳定就业岗位，做好化解过剩产能过程中职工分流安置工作。扎实开展就业援助专项行动，做好城镇失业人员、“4050”人群等群体就业工作，托底帮扶就业困难人员。实施农民工技能提升计划，促进农村富余劳动力转移就业，转移农村劳动力3.1万人。实施职业培训全覆盖计划，推行终身职业技能培训制度，鼓励大学生灵活就业、网络创业，加强失业人员再就业培训，支持返乡农民工创业和引导就近就地就业，提升劳动者就业创业能力和职业转换能力。

　　努力增加城乡居民收入。健全企业工资决定和正常增长机制，发布年度企业工资指导线，促使各类企业职工特别是一线职工能够享受到发展成果。提高退休人员基本养老金标准，确保按时足额发放。全面落实司法体制改革试点工资政策。围绕农民持续增收，推进农业提质增效，努力增加就业岗位和创业机会，着力增加农民经营性收入和工资性收入；深化农村产权制度改革，增加农民财产性收入；全面落实国家和省各项强农惠农补贴政策，增加农民转移性收入，确保农民收入增长实现“三个快于”目标。健全农民工工资支付保障机制，构建和谐劳动关系。

　　全面提升社会保障水平。推动机关事业单位养老保险制度改革入轨运行。实现城乡居民基本医保制度并轨，推进基本医保异地结算和医保支付方式改革，实现异地就医住院费用直接结算。城乡居民医保财政补助由每人每年420元提高到450元。做好低保政策和扶贫政策的有效衔接，保障低保户、五保户纳入建档立卡范围，继续提高农村低保标准，实现全市农村低保标准达到现行国家扶贫标准。全面实施全民参保登记。实施残疾人精准康复服务行动。加大医疗救助和临时救助帮扶力度，落实特困人员救助供养政策，完善困难残疾人生活补贴和重度残疾人护理补贴制度，完善“三留守”关爱保护机制。推进市老年公寓建设前期工作，完成代县、繁峙县、宁武县老年公寓主体工程，开展医养结合试点工作，不断满足老年人健康养老服务需求。

　　积极推进健康忻州建设。深化县级公立医院改革，推进城市公立医院改革，落实政府办医责任，完善医院补偿机制，动态调整医疗服务价格，全部取消药品加成。投运市儿童医院。落实社会办医与公立医院同等待遇，支持有条件的非公立医疗机构做大做强。加快建立分级诊疗制度，提升基层医疗卫生机构服务能力，做实繁峙县县乡医疗卫生一体化试点工作，争取县乡医疗卫生一体化工作上半年在全市推开。开展基本公共卫生服务精细化管理年活动，大力推进基本公共卫生服务网格化管理、签约式服务、责任制指导、“三统一”考核。以建机制为重点，努力在分级诊疗制度、现代医院管理制度、全民医保制度、药品供应保障制度、综合监管制度5项基本医疗卫生制度建设上取得新突破。提高基本公共卫生服务财政补助标准。推动中医药传承、创新、发展。完善全面二孩政策体系。推进全民健身和全民健康深度融合，巩固爱国卫生运动成果。做好第二届全国青运会忻州赛区筹备工作。

　　切实办好民生实事。认真办好省政府确定的6件民生实事，实施2773名残疾预防重点干预和残疾儿童抢救性康复项目，对11个贫困县建档立卡的农村妇女免费进行“两癌”检查，为全市城乡怀孕妇女提供免费产前筛查和诊断服务，新建60个农村老年人日间照料中心，实施1.55万名建档立卡农村贫困劳动力免费职业培训，免费送戏下乡400场。结合忻州实际，抓住具体事情，立说立办。继续办好“周末大戏台”，组织实施好广播电视“户户通”和“一村一月一电影”放映工程。在云中河景区、体育广场设立国民体质监测中心，指导科学健身。进一步方便市民出行，加快忻州城区公交总站建设，开通城区新建道路公交线路和神池、静乐、代县、繁峙县城环保公交车；深化出租汽车行业改革，规范网络预约出租汽车经营服务管理；适时推出城市公交自行车。加强小型游园、广场建设，加快城区市容环境卫生和便民市场等服务设施建设，满足市民生活、休闲需求。改善群众居住条件，城市棚户区改造3830套（户）。切实规范物业管理，让各类群体各得其所，让业主享受到良好服务。开展“维权护农”，在30%的农村建立消费者投诉站和12315联络站。

　　积极发展妇女儿童、老龄、慈善和红十字会等事业，切实加强气象、地震、人防、科普、档案、民族宗教、外事、侨务、双拥等工作，支持国防建设，全面推进基层民主政治建设，依法顺利完成第十一届村民委员会换届选举。

　　（十）狠抓安全生产和生态环保，在持续增强群众生活满意度和幸福感上下功夫。

　　全面加强安全生产工作。牢固树立总体安全观，坚持“党政同责、一岗双责、企业（单位）主责、失职追责”，按照“三个必须”和谁主管谁负责的要求，压实各级政府领导责任、部门监管责任和企业主体责任。推进依法治安，全面落实新修订的《山西省安全生产条例》，严格落实“四铁要求”，坚持标本兼治、综合治理、系统建设，深入落实“4438”机制，完善政府安全监管执法体制，充实安全生产监管执法人员，强化乡镇（街道）执法力量，完善开发区、工业园区、风景区等功能区的安全生产监管体制。进一步健全安全生产“黑名单”制度，严格责任倒查制度，强化事故问责，实行目标考核“一票否决”。完善人防、技防、物防等风险防控措施，全面开展安全风险预控和隐患管理，深入开展安全生产隐患大排查大整治，加大对去产能企业、长期停工停产矿井和技改矿井的检查力度，及时消除各类事故隐患，坚决打击取缔非法生产经营建设行为。强制推进重点领域安全生产标准化建设，强化企业生产一线、作业现场的安全生产管理，坚决杜绝重特大事故、有效遏制较大事故、着力减少一般事故。

　　大力推进生态文明建设。坚持标本兼治，统筹抓好源头治理、过程治理、重点治理、专项治理和系统治理，深入打好大气、水、土壤污染防治“三大战役”，努力改善环境质量。坚决打好蓝天保卫战，开展大气污染防治专项整治攻坚行动，加大以雾霾为重点的环境污染治理力度，引深“铁腕治污”行动，依法严惩各类环境违法违规行为，坚决把“三不要”“三要”要求落到实处。深化“控煤、治污、管车、降尘”重要举措，全部淘汰忻州城市建成区燃煤小锅炉，推进冬季清洁取暖，推进以电代煤、以气代煤；加大燃煤电厂超低排放和节能改造力度，开展重点行业污染治理专项行动，对所有重点工业污染源实行24小时在线监控，到期不达标的坚决依法关停；强化机动车尾气治理，基本淘汰黄标车，加快淘汰老旧机动车。深入推进汾河、滹沱河、桑干河等重点流域生态修复治理，全面推行“河长制”。强化森林资源管护，力度不减推进造林绿化，完成营造林80万亩以上。

　　全力维护社会稳定大局。发挥基层组织作用，深化社区“网格化”管理，完善城乡社区、社会组织、社会工作联动机制。完善重大决策社会稳定风险评估机制，重视公众心理疏导，加强对社会热点难点问题的引导，解决好涉及群众切身利益的问题。加强食品药品监管，建立重要产品追溯体系。营造良好金融生态环境，严厉打击恶意逃废金融债务和各类非法金融活动，坚决防止发生区域性、系统性金融风险。深化平安忻州建设，狠抓社会治安综合治理，严厉打击违法犯罪，努力营造和谐稳定的社会环境。

　　三、改进政府工作

　　严守法治底线。强化法治意识和法治思维，全面推进依法行政。坚持科学决策、民主决策、依法决策，实行重大行政决策合法性审查机制、终身责任机制。自觉运用法治思维和法治手段解决问题、推动工作，确保政府一切行为在法律和政策范围内活动，确保每项工作经得起法律的审查和历史的检验。设立政府法律顾问和公职律师。加强审计监督，强化巡察、检察对行政权力的监督和制约。扎实开展“七五”普法，加强法律援助。

　　紧紧依靠群众。坚持以人民为中心的发展思想，把群众拥护不拥护、赞成不赞成、高兴不高兴、答应不答应作为衡量政府一切工作得失的根本标准，努力实现好、维护好、发展好最广大群众的根本利益。坚持从群众中来，到群众中去，深入一线，听取企业家、基层干部和普通群众的意见，问计于群众、问需于群众，确保各项决策部署顺应群众意愿，切合发展实际。尊重群众主体地位，加强教育引导，打通思想，转变观念，让广大群众心甘情愿支持政府的工作，切实把群众的行动、热情、活力凝聚到促进忻州转型升级、振兴崛起上来。

　　加快职能转变。处理好新常态下政府与市场、企业、群众、社会的关系，充分发挥好市场在资源配置中的决定性作用，更好发挥政府作用，切实管好政府该管的事。推动“放管服效”改革向纵深发展，继续推进简政放权，努力做到审批最少、流程最优、体制最顺、机制最活、效率最高、服务最好。开展相对集中行政许可的试点，加快推进投资审批网上办理和并联审批。推进“互联网+政务服务”，提升“两平台”服务功能，建设全市政务服务“一张网”，实现服务事项“一号申请、一窗受理、一网通办”，进一步提升行政效能。普及网络知识、推动网络建设，实现宾招机关、车站广场等公共场所“WIFI”免费全覆盖。

　　提升工作效能。推进智慧政府建设，强化资源整合，探索运营机制，实行政府购买服务，进一步提升信息化服务水平。拓展“忻州随手拍”的作用，持续加大网络问政力度，推动网络问政常态化；筹备成立融媒体中心，深化拓展延伸“13710”工作制度，建立政府系统抓落实的刚性约束和长效机制，探索纪检委适时入驻“13710”系统制度，实行重大决策部署、重要改革事项、重点工作任务清单化管理，推动政府各项工作见人见事、见根见果，抓细抓小、抓紧抓实。持续提升政府工作人员了解规则、掌握规则、运用规则的能力，把握实际情况，抢抓政策机遇，始终站在法律和政策的制高点。健全重点工作督查机制，强化督查职能，盯住关键环节，确保各项工作任务全面及时高效落实。推进追责问责制度化，对不作为、慢作为、不落实、假落实行为，严肃追责问责。

　　大力从严治政。严格落实全面从严治党要求，履行好政府党组落实全面从严治党任务的主体责任，严格执行党内政治生活准则，继续在常和长、严和实、深和细上下功夫。始终把纪律和规矩挺在前面，深入推进政府系统党风廉政建设和反腐败斗争，推动落实中央八项规定和国务院“约法三章”常态化、长效化；持续狠刹“四风”，厉行勤俭节约，继续压减“三公”经费和一般性支出。在财政继续减收的背景下，继续增加民生支出，切实把有限的资金用在改善民生上。注重政府干部的心理疏导和健康教育，政治上关怀、思想上关心、生活上关爱，以良好精神状态应对繁重复杂的任务。

　　自觉接受监督。自觉接受人大及其常委会的监督，主动接受政协的民主监督，认真听取民主党派、工商联、无党派人士和人民团体的意见，重视司法、舆论、社会监督，广泛接受群众监督，让权力在阳光下运行。全面推行和细化“双随机、一公开”监管，充分发挥传统媒体、新兴媒体作用，强化问题跟踪督办，积极回应社会关切。

　　各位代表，转型综改、创新驱动，顺应发展潮流，符合忻州实际，是忻州振兴崛起、全面脱贫、全面小康的制胜之道、光明大道。无论这条路走起来多么艰难，我们都要坚定不移地走下去，直至达到胜利的彼岸。我们的事业是正义的，正义的事业，任何艰难险阻都不可阻挡。让我们更加紧密地团结在以习近平同志为核心的党中央周围，在市委领导下，全面实施“1661”发展战略，紧紧扭住发展第一要务，认真坚持“产业第一、项目至上、企业为重、服务为本”，忠诚担当、开拓创新、攻坚克难、奋发图强、不忘初心、继续前进，以实际行动和崭新成绩迎接党的十九大胜利召开！
